# Neuroimaging Study Designs, Computational Analyses and Data Provenance Using the LONI Pipeline

**DOI:** 10.1371/journal.pone.0013070

**Published:** 2010-09-28

**Authors:** Ivo Dinov, Kamen Lozev, Petros Petrosyan, Zhizhong Liu, Paul Eggert, Jonathan Pierce, Alen Zamanyan, Shruthi Chakrapani, John Van Horn, D. Stott Parker, Rico Magsipoc, Kelvin Leung, Boris Gutman, Roger Woods, Arthur Toga

**Affiliations:** 1 Laboratory of Neuro Imaging, University of California Los Angeles, Los Angeles, California, United States of America; 2 Department of Computer Science, University of California Los Angeles, Los Angeles, California, United States of America; Indiana University, United States of America

## Abstract

Modern computational neuroscience employs diverse software tools and multidisciplinary expertise to analyze heterogeneous brain data. The classical problems of gathering meaningful data, fitting specific models, and discovering appropriate analysis and visualization tools give way to a new class of computational challenges—management of large and incongruous data, integration and interoperability of computational resources, and data provenance. We designed, implemented and validated a new paradigm for addressing these challenges in the neuroimaging field. Our solution is based on the LONI Pipeline environment [3], [4], a graphical workflow environment for constructing and executing complex data processing protocols. We developed study-design, database and visual language programming functionalities within the LONI Pipeline that enable the construction of complete, elaborate and robust graphical workflows for analyzing neuroimaging and other data. These workflows facilitate open sharing and communication of data and metadata, concrete processing protocols, result validation, and study replication among different investigators and research groups. The LONI Pipeline features include distributed grid-enabled infrastructure, virtualized execution environment, efficient integration, data provenance, validation and distribution of new computational tools, automated data format conversion, and an intuitive graphical user interface. We demonstrate the new LONI Pipeline features using large scale neuroimaging studies based on data from the International Consortium for Brain Mapping [5] and the Alzheimer's Disease Neuroimaging Initiative [6]. User guides, forums, instructions and downloads of the LONI Pipeline environment are available at http://pipeline.loni.ucla.edu.

## Introduction

The success of contemporary computational neuroscience depends on large amounts of heterogeneous data, powerful computational resources and dynamic web-services [7]. Advanced neuroimaging studies require multidisciplinary expertise to construct complex experimental designs using diverse data, independent software tools and disparate networks. Design and validation of analysis protocols are significantly enhanced by graphical workflow interfaces that provide high-level manipulation of the analysis sequence while abstracting many implementation details.

High-throughput analysis of large amounts of data using scripting or graphical interfaces has become prevalent in many computational fields, including neuroimaging [4], [8], [9]. Fundamental driving forces in this natural evolution of automation are massive parallelization, increased network bandwidth, and wide distribution of efficient and robust computational and communication resources. Rapid increases in resource development and their wide utilization enable the expansion of integrated databases and vibrant human/machine communications, as well as distributed grid and network computing [10], [11]. This manuscript presents the visual language programming of study designs and data provenance based on complex pipeline workflows using the LONI Pipeline environment. We demonstrate the construction, validation and dissemination of study designs via the LONI Pipeline using advanced neuroimaging protocols analyzing multi-subject data derived from the International Consortium for Brain Mapping (ICBM) [5] and the Alzheimer's Disease Neuroimaging Initiative (ADNI) [6].

There is a significant variation and a continual evolution among different groups, investigators and research sites in the development styles, design specifications and analysis protocols of newly engineered resources. To provide an extensible framework for interoperability of these resources the LONI Pipeline employs a decentralized infrastructure, where data, tools and services are linked via an external inter-resource mediating layer. Thus, no modifications of the existing resources are necessary for their integration with other computational counterparts. The Pipeline eXtensible Markup Language (XML) schema forms the backbone for the inter-resource mediating layer. Each XML resource description includes important information about the resource location, the proper invocation protocol (i.e., input/output types, parameter specifications, etc.), run-time controls and data-types. This XML schema also includes auxiliary metadata about the resource state, specifications, history, authorship, licensing, and bibliography. The LONI Pipeline infrastructure (http://pipeline.loni.ucla.edu) facilitates the integration of disparate resources and provides a natural and comprehensive data provenance [12]. It also enables the broad dissemination of resource metadata descriptions via web-services and the constructive utilization of multidisciplinary expertise by experts, novice users and trainees.

### 1.1 Current approaches for software tool integration and interoperability

**Table 1** summarizes various efforts to develop environments for tool integration, interoperability and meta-analysis [13]. There is a clear need to establish tool interoperability as it enables new types of analyses, facilitates new applications, and promotes interdisciplinary collaborations [14]. Compared to other environments, the LONI Pipeline offers several advantages, including a distributed grid-enabled client-server infrastructure and efficient deployment of new resources to the community: new tools need not be recompiled, migrated or altered to be made functionally available to the community.

**Table 1:** Comparison between the LONI Pipeline v.4 and other existing environments for software tool integration and interoperability.

Name/URL	Community-Based Resource Development	Requires Tool Recompiling	Data Storage	Platform Independent	Client-Server Model	grid Enabled	Application Area	Citations
LONI Pipeline v.4pipeline.loni.ucla.edu	Y	N	External	Y	Y	Y (DRMAA API)	Neuroscience	140(v.4)
Taverna [54]	Y	Y (via API)	Internal (MIR)	Y	Y	Y (*^my^*GRID)	Bioinformatics	652
Kepler [70]kepeler-project.org	Y	Y (via API)	Internal (actors)	Y	N	Y (Ecogrid)	Area agnostic	414
Triana [71]trianacode.org	Y	Y	Internal data struct	Y	N	Y (gridLab)	Heterogeneous Apps	120
Pegasus [72]pegasus.isi.edu	Y	Y	External	Y	N	Y(Globus CondorG)	Heterogeneous Apps	386
SCIRun [73]software.sci.utah.edu	Y	Y (via API)	Internal	N	Y	N	Image processing	177
Slicer [74]slicer.org	Y	Y (via API)	Internal	N	Y	N	Medical imaging	139
MediGRID [75]medigrid.de	Y	Y	N/A	Y/N	N	Y	Biomedical	32
Khoros [76] www.khoral.com	N	Y (via API)	Internal	N	Y	Y	Imaging Processing	118
MAPS [77] http://iacl.ece.jhu.edu	Y	Y	Internal	Y	N	N	Brain imaging	10
OpenDX [78] www.opendx.org	Y	N (requires tool data wrapper)	Internal	Y	Y	N	Heterogeneous Apps	35
SWIFT [26]ci.uchicago.edu/swift	Y	N	Internal or External	Y	Y	Y (Globus)	Area Agnostic	24
Trident Workbench [79]	N	Y(C#/MFC/WWF)	Internal	N	Y	Y(HPC Cluster)	Oceanography	5
Karma2 [80]	N	Y	Internal	Y	Y	N	Imaging	11

The *citation* column contains the number of citations of the main publication for each tool (as of March 2010), according to Google Scholar.

Any comparison between these and other workflow environments will show strengths and weaknesses within each. The choice of workflow infrastructure often depends on the application domain, the type of user, types of access to resources (e.g., computational framework, human or machine resource interface, database, etc.), and the desired features and functionalities [15]. The inevitable similarities between the LONI Pipeline and other such environments include the graphical interfaces to enable the design and improve usability of the analysis protocols. Visual interfaces present complex analysis protocols in an intuitive manner and improve the management of technical details. Most graphical workflow environments also provide the ability to save, load and distribute workflows through servers using Simple Object Access Protocol (SOAP), Web-services Description Language (WSDL), XML, or other protocols [9].

The LONI Pipeline differs from many of the other environments in that it was developed with imaging computations in mind for general neuroscience users, and its goals include portability, transparency, intuitiveness and abstraction from grid mechanics. The Pipeline is a dynamic resource manager, treating all resources as well-described external applications that may be invoked with standard remote execution protocols. The LONI Pipeline XML description protocol allows any command-line driven process, web-service or data-server to be accessed within the environment *by reference*. This means that the actual data, tools and services included in pipeline workflows are referenced by locations and not imbedded in the Pipeline environment itself. At workflow-execution time, all references and dependencies are checked and validated dynamically. There is no need to reprogram, revise or recompile external resources to make them usable within the LONI Pipeline. This design reduces the integration costs of including new resources within the LONI Pipeline environment and provides the benefit of quick and easy management of large and dispersed data and resources. In addition, this choice significantly reduces the hardware and software requirements (e.g., memory, storage, CPU) on the user/client machine. Finally, a key difference between the LONI Pipeline and most other environments is its management of distributed resources via its client-to-server infrastructure and its ability to export automated makefiles/scripts. These allow the LONI Pipeline to provide processing power independently of the available computational environment (e.g., operating systems, grid, mainframe, desktop, etc.) [16]. The LONI Pipeline servers communicate and interact with Pipeline clients and facilitate secure transfer of processes, instructions, data and results via the Internet. The LONI Pipeline also simplifies the inclusion of external data display modules and facilitates remote database connectivity such as the LONI Imaging Data Archive (ida.loni.ucla.edu) and the Extensible Neuroimaging Archive Toolkit (www.xnat.org) [17].

The core LONI Pipeline functionality is based on our prior experience [3], user feedback and information technology advancements over the past several years. The current LONI Pipeline functionality includes a tool discovery engine, a plugin interface for meta-algorithm design, a grid interface, secure user authentication, data transfers and client-server communications, graphical and batch-mode execution, encapsulation of tools, resources and workflows, and data provenance.

### 1.2 Types of tools and services that can be integrated within the LONI Pipeline

The development and utilization of the LONI Pipeline environment is focused on neuroimaging data and analysis protocols. However, by design, the LONI Pipeline software architecture is domain agnostic and has been adopted in other research and clinical fields, e.g., bioinformatics [14]. There are two major types of resources that may be integrated within the LONI Pipeline. The first one is *data*, in terms of databases, data services and file systems. The second type includes stand-alone *tools*, comprising local or remote binary executables and services with well-defined command line syntax. This flexibility permits efficient resource integration, tool interoperability and wide dissemination. Neuroimaging tools developed at the Laboratory of Neuro Imaging represent a fraction of all the tools which have been wrapped in XML module/pipeline descriptions. Examples of resources developed at other institutions that are already available within the Pipeline include FSL/Oxford [18], Freesurfer/Harvard [19], AFNI/NIH [20], XNAT/BIRN [17], MNI/McGill [21], etc.

### 1.3 Core Pipeline Functionality, Features and Interfaces

The Pipeline environment aims to provide a user-friendly mechanism for designing analysis protocols as complete neuroimaging studies starting with raw imaging data and metadata, and ending with quantitative and interpretable results. The most notable Pipeline features include:

#### 1.3.1 Scalability

The Pipeline environment provides scalability of data analysis on several levels. The processing time scales directly with the number of subjects included in the analysis protocol and is inversely proportional to the number of compute nodes. The Pipeline's task-manager provides load-balancing and user-management, and integrates the direct and batch processing capabilities of available grid-management environments such as Oracle Grid Engine (OGE, http://www.oracle.com/us/products/tools/oracle-grid-engine-075549.html) and Torque (www.clusterresources.com). For example, when running the OGE Java grid engine Database Interface (JGDI) plugin, the Pipeline server receives asynchronous events when an Oracle Grid Engine job for given user is complete. Because there is no polling involved, the Pipeline server can efficiently track the status of a large number of workflows and jobs per workflow. The Pipeline also provides virtualization, robustness features like rerun/troubleshooting, functionality to optimize processing protocols, and user specification of grid parameters, tools usage statistics and fair-usage policies.

#### 1.3.2 Plugin Extensions

The Pipeline plugin application programming interface (API) allows two layers of lightweight extensions. These include server restartable plugins of the backend and grid components, as well as client-level plugins that allow interlacing local processing within complex pipeline workflows (e.g., LONI Viewer for data visualization).

Consider the problem of connecting the Pipeline server to distributed resource managers supporting a variety of APIs. These resource managers and APIs may have advantages and disadvantages. For example, the Distributed Resource Management Application API (DRMAA), www.drmaa.org, is standardized and supported by several resource managers. However, it does not facilitate job execution and tracking based on the user id of the user who is running the workflow that contains the given job. The internal JGDI interface provides scalable event and user ID based tracking of job execution status. The Pipeline server plugin system allowed the development of both a DRMAA and JGDI plugin interfaces. The Pipeline runs the more efficient JGDI plugin when the server connects to a backend OGE resource manager. The Pipeline architecture provides a DRMAA plugin for connecting to other resource managers. Planned future developments include Pipeline server plugins for gLite (http://glite.web.cern.ch/), SAGA (http://saga.cct.lsu.edu/) and other APIs.

The DRMAA and JGDI plugins, described above, run in the same operating systems process as their associated Pipeline server. However, this is not a requirement for all server side plugins. In our efforts to provide a high degree of fault tolerance and availability for the Pipeline service, we developed a restartable JGDI plugin that runs in a separate process. A restartable plugin may receive signals (e.g., halting, stopping, terminating) from the operating system, which may cause a crash of the plugin. However, the plugin acts as fault isolation layer for the Pipeline server and protects the Pipeline server process from receiving and processing such potentially critical signals. The Pipeline server detects when plugins crash and restarts them as appropriate. Restartable plugins and plugins running inside the Pipeline server-process both support the same Java API.

#### 1.3.3 Smartlines

In heterogeneous processing workflows, smartlines are a new user-friendly feature that facilitates the automated conversion, formatting and transfer of data between provider and receiver modules. Often, data-types, data-formats and data-representation of the same information may vary between executable modules in a pipeline workflow, especially when a heterogeneous workflow includes tools developed by different groups and for different purposes. This presents a challenge for many novice users. One approach is to include data-converter modules between data provider and receiver modules, which explicitly convert and feed the data from one module to the next. The new smartlines approach implicitly converts different data types and formats according to the specification of the provider and receiver modules. The current LONI Pipeline smartlines automatically convert between the common neuroimaging data types (e.g., Analyze, MNC, Nifti, MGZ, raw, byte, short, float, etc.) [22]. The smartline plugin infrastructure enables the extension of these file-conversions to ensure the smooth flow of diverse data and parameters throughout pipeline workflows. To abstract the data type and format issues, the smartlines use the LONI Debabeler [23] to convert files based on the XML definitions of the source (provider) and target (receiver) modules. Just like other Pipeline modules, Smartlines are executed in parallel, automatically identify the input file formats, inspect the module metadata, and check the image data types against the appropriate (input) types in the target Pipeline module. Smartlines have a distinct appearance (shape and color), they are implemented in pure Java, and use the LONI Debabeler plugin architecture to enable easy addition of new file types. Adding a new smartline conversion requires using the Debabeler GUI interface to edit the current smartline translations. Smartlines do not fully distance the user from the conversion process when user input is needed (e.g., how to down-sample an image).

#### 1.3.4 Grid Monitor

The LONI Pipeline environment also includes an interactive grid monitor – a graphical plugin widget that allows real-time web-service-based inspection of the status of the background computational grid. The three-dimensional visualization gives users a quick, intuitive feel for the current usage of the cluster; each bar on the ring represents a single execution node, and the height of each bar represents that node's CPU load. To add further clarity, the center of the ring shows the numerical ratio of active to inactive CPU core counts, and on the left and right sides are statistical plots of total cluster core and network usage. This service can even be viewed completely independently of the Pipeline client by directly accessing the following Java applet http://www.loni.ucla.edu/Resources/clustervisualization/. The grid Monitor adds another layer of ease-of-use to the Pipeline service; users can, within less than a minute, easily see whether they should expect to execute immediately or following queuing delays, and can adjust their submission times and/or schedules accordingly.

### 1.4 LONI Pipeline Graphical and Scripting Interfaces

Pipeline workflows (.pipe files) may be constructed in many different ways (e.g., using text editors) and these protocols may be executed in a batch mode without involving the LONI Pipeline graphical user interface (GUI). However, the GUI significantly aids most users in designing and running analysis workflows. A library of available tools is presented in the left panel of the LONI Pipeline client window. Users may search for, drag and drop these tools onto the main canvas to create or revise a workflow. Connections between the nodes represent the piping of output from one program to another. This is accomplished without requiring the user to specify file paths, server locations or command line syntax. Pipeline workflows may be constructed and executed with data dynamically flowing (by reference) within the workflow. This enables trivial inclusion of pipeline protocols in external scripts and integration into other applications. Currently, the LONI Pipeline allows exporting of any workflow from XML (*.pipe) format to a makefile or a shell script for direct or queuing execution.

### 1.5 Pipeline Usability

The new version 5.0 of the LONI Pipeline improves a number of usability features. These include the editing and usage modes of the graphical user interface, application state-specific menus and help widgets, pop-up and information dialogs, the handling of local and global variables within the pipeline, the integration of data sources and executable module nodes, data type checking and workflow validation, client connect and disconnects, job management and client-server communications.

The Pipeline supports workflow *pause* and *resume* functions. While a workflow is executing, the user can pause the workflow by clicking the Pause button. All running jobs in the workflow will be stopped and all their output files will be deleted. Outputs from already completed modules will be preserved and available for subsequent use. The Pipeline server saves the paused workflow status into its persistent database so users can disconnect from the server and later retrieve and explore the state of the workflow. Any paused workflow can be restarted by the user, and the Pipeline server will resume the workflow execution from the paused state. The pause and resume features give users more flexibility in managing the workflow control. The Pipeline *restart* function is available for any (normally or erroneously) completed module in a workflow. When a user restarts a module, all instances for this module, and its successor modules, will be resubmitted to the server for execution. To avoid possible conflict following a restart, all subsequent output files will be deleted. Parent modules and modules from other independent branches in the workflow will not be affected by a restart. This restart functionality is useful for debugging and troubleshooting complex workflow protocols and saves time by avoiding execution of downstream modules.

The pipeline *validation* feature offers interactive support for running or modifying existing pipeline workflows. This feature checks the consistency of the data types and parameter matches, validity of the analysis protocol, and schedules module execution. The LONI Pipeline intelligence component reduces the need to review details or double check modifications of new or existing workflows. Still, users control the processes of saving workflows and module descriptions, data input and output, and the scientific design of their experiments. This functionality significantly improves usability and facilitates scientific exploration.

## Methods

We developed an infrastructure that enables integration of individual executable software programs (tools or modules) and web-services (e.g., database services) into a graphical processing pipeline (workflow). Each pipeline contains a complete description of its component modules/services, the necessary connectivity information between processing modules and the module control parameters appropriate for its specific execution. The LONI Pipeline infrastructure enables communication of files, data, control parameters and intermediate results between the modules [3]. Neither the computational tools nor the data are stored internally within the LONI Pipeline environment. Only object references are stored in XML format and are appropriately passed between inter-connected modules within the pipeline workflow. This infrastructure allows direct workflow encapsulation where a pipeline network may be contained and utilized as a module within a subsection of another pipeline workflow. This conceptual abstraction layer facilitates the construction, revision and utilization of analysis workflows by expert, general and novice users. The LONI Pipeline execution environment controls the local and remote server connections, module communication, process management, data transfers and grid mediation. The XML descriptions of individual modules, or networks of modules, may be constructed, edited and revised directly within the LONI Pipeline graphical user interface, as well as saved or loaded from disk, URL or the LONI Pipeline server. These workflows completely describe new methodological developments and allow validation, reproducibility, provenance and tracking of data and results.

### 2.1 Study-Design Interface

**Figure 1:**
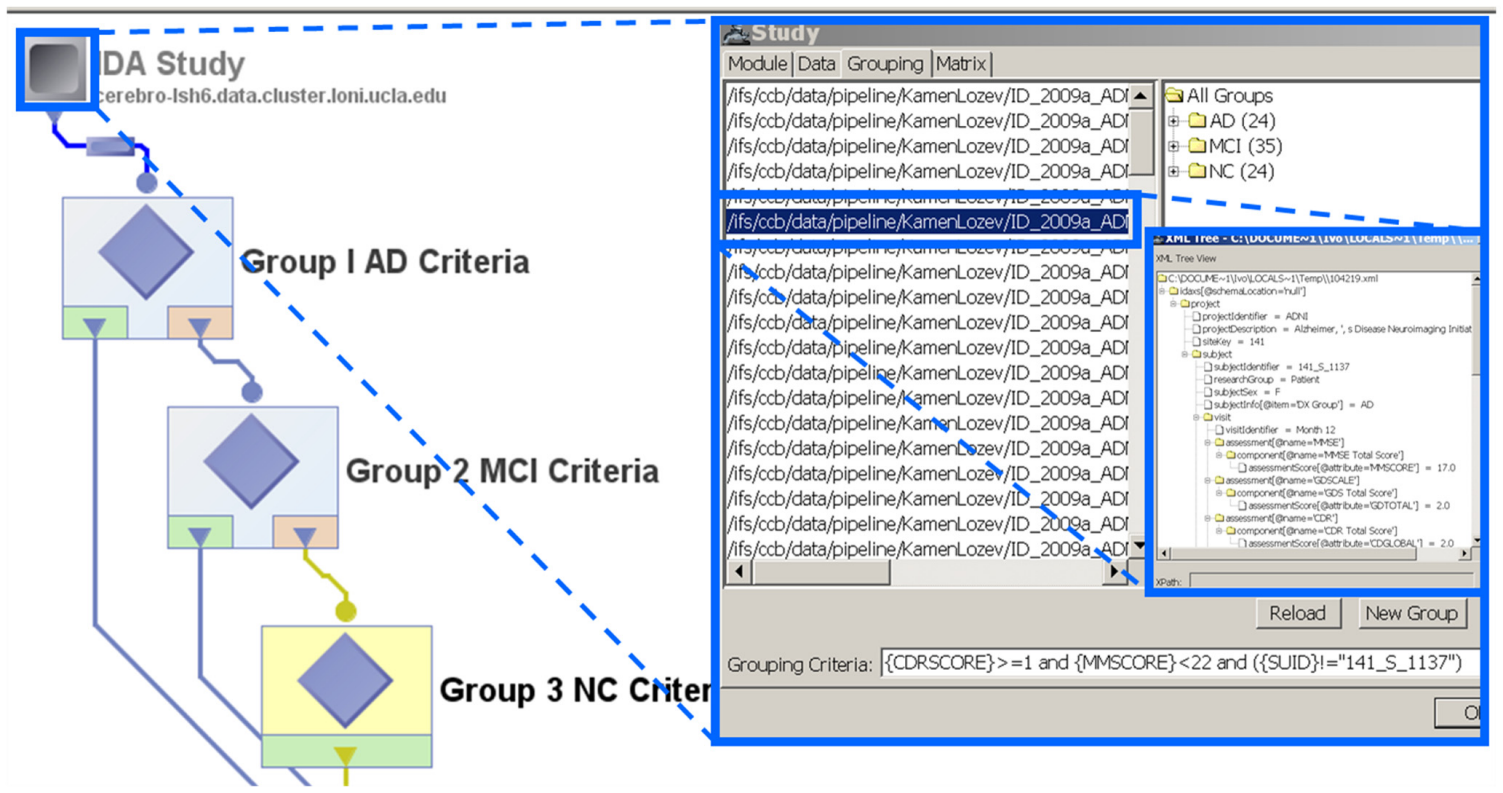
Pipeline Study-Design Architecture. Imaging and non-imaging meta-data for 104 subjects is used to stratify the entire population into 3 distinct cohorts – asymptomatic normal controls (NC), Alzheimer's disease (AD) patients, and Mild Cognitive Impairment (MCI) subjects. The nested inserts show the search and selection grouping criteria, cohort sizes and an instance of an XML meta-data file for one subject. The meta-data can be manually entered, automatically parsed from spreadsheets, databases or clinical charts, or fed in as results of the pipeline workflow calculations (derived data).

The Study-Design interface enables the integration of imaging data and supporting metadata. Imaging data is any data modality where univariate intensities, or higher-dimensional vectors or tensor attributes, are stored on a regular matrix (multidimensional grid or lattice). The matrix grid frequently corresponds to space-time dimensions and may represent isotropic or non-isotropic hypervoxels – the higher-dimensional analogues of 2D pixels. The metadata contains tabular clinical, demographic and phenotypic data linked to and supporting the imaging data, such as subject demographics (e.g., age, gender), scanning parameters, clinical scores, and other observational measures about each subject. The metadata represents measurements which are not uniformly available for each space-time point (voxel) the way the imaging data are. The fundamental representation differences between imaging and non-imaging metadata introduce a significant challenge in integrating and analyzing the complete dataset within subjects and across populations. The study-design protocol addresses this challenge by enabling the stratification of complex groups and cohorts based on both imaging and non-imaging data. These cohorts may then be modeled individually, or compared against each other, according to the research hypotheses put forward by the investigators before the data collection. The Pipeline study-design functionality facilitates the integration of imaging and non-imaging data available in disparate databases, file-systems and spreadsheet formats. **Figure 1** illustrates the main states of study-design construction, selection and study groups generation.

A study-design module is similar to a data source and can be connected to the input parameters of other modules in the same workflow. It allows passing both imaging data and the metadata information to subsequent modules and all of this study information may be passed along throughout the pipeline workflow. The metadata may be used for setting up various conditional criteria in conditional modules. The metadata information may be represented as an XML file, as long as its schema is valid (well-formed) and consistent (uniform for every subject in the study), or as a tabular spreadsheet (CSV).

Users may create study-design modules by importing data from directories, by specifying the file paths on local or remote servers, or by importing XML formatted metadata. There are three specific ways to construct study-design modules:

- *Using Filenames/Directories*: Users may specify filename matching rules and root directories to find all files under the root directory that match data and metadata according to the chosen matching rule. Recursive traversal of subdirectories is also allowed. In order to restrict the search to only certain type of data, the type of file option may be used. Filters can also be used to restrict the search based on some criteria.
- *Metadata Import*: Users may also construct study-design modules by specifying metadata rules and a list of metadata files. This enables deriving data paths from XML elements of the metadata and subsequent matching of the metadata with the derived data. In order to do this, a directory path that contains these metadata files and the element name that contains the data path has to be specified. Again, recursive filename matching rules may be specified to traverse and filter appropriate files and content.
- *Spreadsheet Import*: The last type of study-design construction uses tabular CSV metadata that contains a list of subject metadata information in a special format. The first row in this file corresponds to the column names/headings. Any information that is required about the subject could be listed in each column. The paths to the imaging data file for each subject must always be listed within one of the columns. Starting from the second row down, the specific metadata for each subject is included, one subject per row. The Pipeline reads the CSV file and automatically creates one hierarchical XML metadata file for each subject and links these metadata files with the paths to the corresponding imaging data.

The complete details and examples of study-design module functionality and utilization is available online (http://pipeline.loni.ucla.edu/support/user-guide/building-a-workflow/#Study).

### 2.2 Database Interface

**Figure 2:**
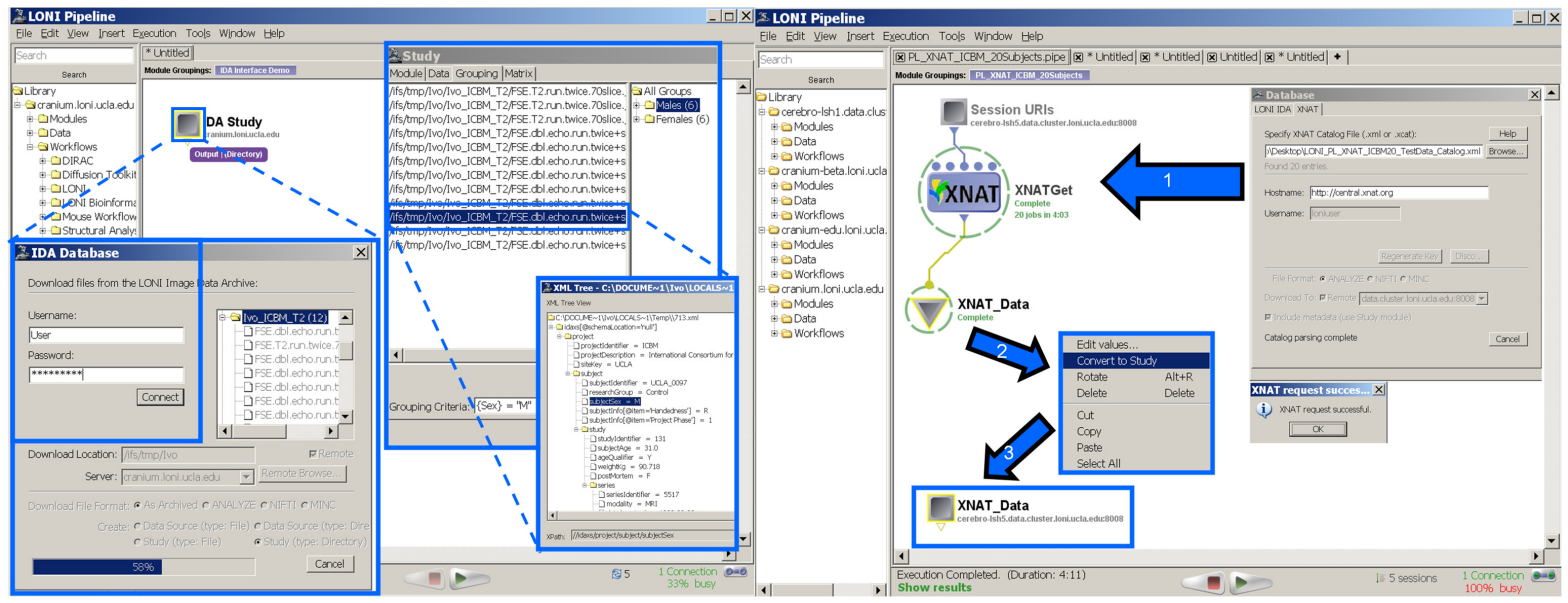
Examples of the Pipeline Database Plug-in Infrastructure using the Imaging Data Archive (IDA) database (left) and the XNAT database (right). Secure user-authentication provides an appropriate data-access level. The user then selects a location for local/remote storage of the data for the computational Pipeline processing and a format for the data representation (e.g., study-design). The data-download progress monitor provides information about the status of the transfer. At the end a study-design, or a data-source module is constructed, which allows the stratification of the population into groups (Male/female, in this case).

The Pipeline environment allows the development of new data input and output parsers for streamlining the protocols of data management and utilization within the Pipeline. Examples of this plugin application programming interface (API) include the Pipeline-Imaging Data Archive (IDA) interface and the Extensible Neuroimaging Archive Toolkit (XNAT) database [17]. This lightweight plugin API architecture supports portals that simplify the data management and transfer between external databases and the pipeline environment. **Figure 2** demonstrates the Pipeline IDA and XNAT graphical user interfaces, which employ secure SSL authentication and allow users appropriate level of access to data archives.

When a user retrieves a collection through the Pipeline database interface, the LONI Pipeline can automatically generate a study design module – e.g., an IDAGet or XNATGet module for retrieving all the neuroimaging data included in the study design module. At execution time, these LONI Pipeline *get* modules are automatically parallelized, as are all other Pipeline server modules with multiple input instances. This enables secure parallel data access and retrieval of large neuroimaging collections stored in external databases. We have observed performance improvements by several orders of magnitude in the download time of large neuroimaging collections as a result of the development of this parallel data access technology. Metadata is retrieved first from the external database, so that study design modeling can begin immediately. The large-volume imaging data can then be retrieved in parallel by the appropriate *get* module and stored into a Study Design module. This interface provides one way for accessing and managing neuroimaging data and metadata and enables easy and efficient import of data collections from external sources into Pipeline study modules.

### 2.3 Visual Programming Language

The Pipeline programming language is a visual environment for expressing large scale parallel computation. The Pipeline environment aims to provide information networking infrastructure for connecting large scale distributed computational and data resources through an information networking stack. The Pipeline programming language is the head of this service stack. Pipeline client and server software provides a language runtime and implements other elements of the service stack such as data transfer and communication with distributed resource managers.

**Figure 3:**
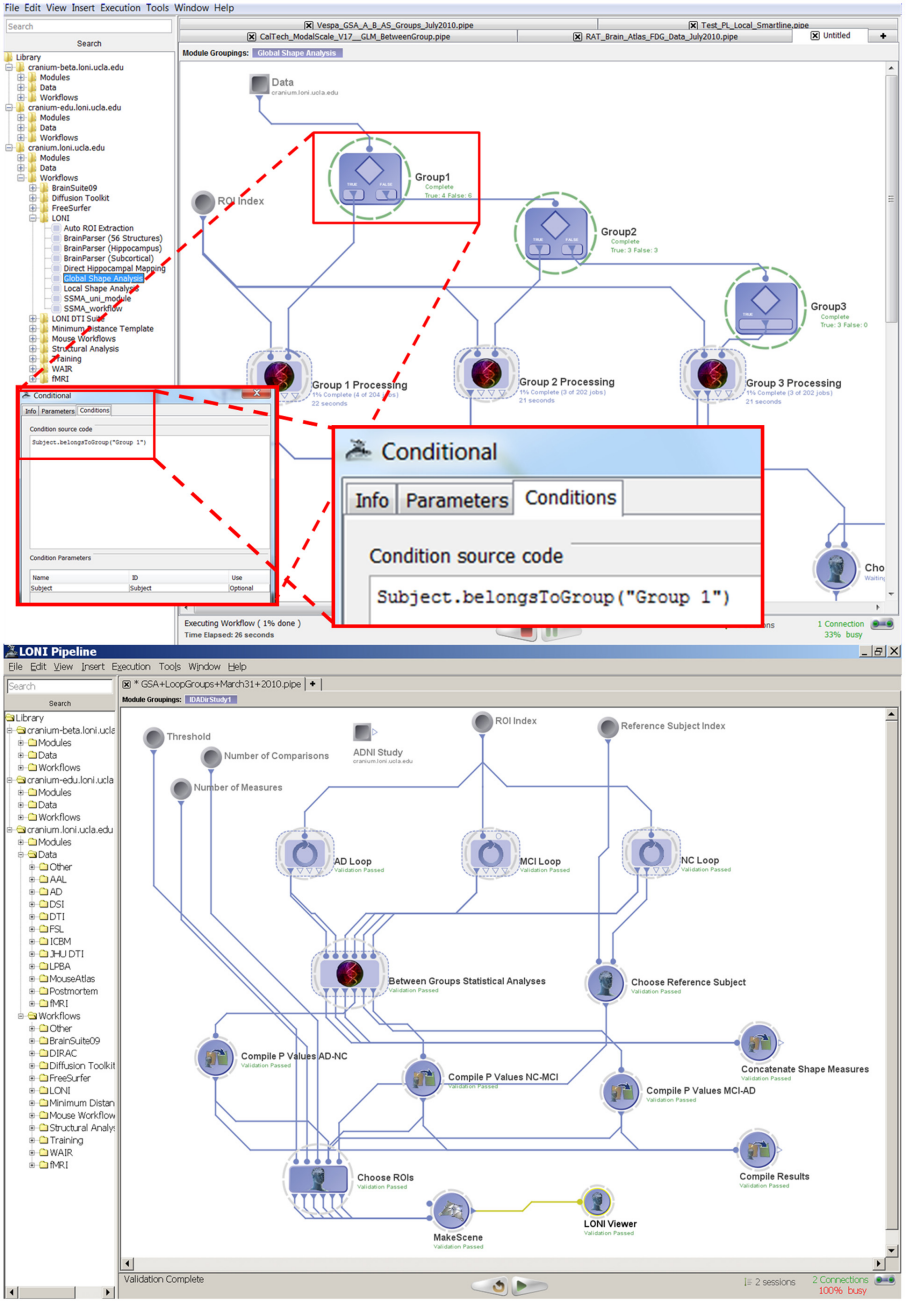
Pipeline environment as a visual programming language. Top panel (**Figure 3.A**) shows an example of a conditional flow of control (if-else), which splits a group of subjects into 3 cohorts, processes each cohort separately and finally maps statistical differences between the 3 groups. The bottom panel (**Figure 3.B**) demonstrates the Global Shape Analysis (GSA) pipeline workflow with efficient loop-group iterations – see the 3 loop-group modules on the top, one for each for the 3 population cohorts (AD, MCI and NC subjects).

The Pipeline environment provides the functionality of a complete graphical programming language. It includes global and local variables, conditional statements, loops/iterators and nested module groups. In addition, the ability to start, pause, restart/continue and stop the workflow execution enables the construction, validation, debugging and on-the-fly modification of complex data-analysis workflows. **Figure 3.A** demonstrates the new conditional flow of control features. In addition, users can treat module inputs or outputs as large arrays and reference the order or index of the current value of a given input or output parameter.

The loop-group implementation provides a one-click group encapsulation of the indexing of the computation. Loop groups enable explicit, dedicated and convenient strategies for expressing single level and nested loops. A Global Shape Analysis (GSA) workflow with loops is illustrated in **Figure 3.B**. In the current implementation of loop groups, the number of loop-body invocations is determined before the loop begins to execute; this allows straightforward parallelization of common cases such as iterating over a known set of images. The LONI Pipeline also provides a simple dynamic repeat until looping functionality at the individual module level.

### 2.4 General LONI Pipeline Computational Infrastructure

The LONI Pipeline routinely executes thousands of simultaneous jobs on our symmetric multiprocessing systems (SMP) and on DRMAA (www.drmaa.org) clusters. On SMP systems, the LONI Pipeline can detect the number of available processing units and scale the number of simultaneous jobs accordingly to maximize system utilization and prevent system crashes. For clusters, a grid engine implementing DRMAA, with Java bindings, may be used to submit jobs for processing, and a shared file system is used to store inputs and outputs from individual jobs. It's possible to extend the LONI Pipeline server plugin to enable job management on other grid infrastructures, e.g., Condor (http://www.cs.wisc.edu/condor/), Globus (www.globus.org), etc.

The LONI Pipeline environment has been integrated with the Pluggable Authentication Module (PAM), to enable a username and password challenge-response authentication method using existing credentials. A dependency on the underlying security and encryption system of the LONI Pipeline server's host machine offers maximum versatility in light of the diverse policies governing system authentication and access control.

Using Java binding to DRMAA interface, we have integrated the LONI Pipeline environment with the Oracle Grid Engine (OGE), a free, well-engineered distributed resource manager (DRM) that simplifies the processing and management of submitted jobs on the grid. It is important to note, however, that other DRMs such as Condor and Torque could be made compatible with the LONI Pipeline environment using the same interface. DRMAA's Java implementation allows jobs to be submitted from the LONI Pipeline to the compute grid without the use of external scripts and provides significant job control functionality internally. We accomplished several key goals with the LONI Pipeline-DRMAA-OGE integration:

1. the parallel nature of the LONI Pipeline environment is enhanced by allowing for both horizontal (across compute nodes) and vertical (across CPUs on the same node) processing parallelization;
2. the LONI Pipeline's client-server functionality can directly control a large array of computational resources with DRMAA over the network, significantly increasing its versatility and efficacy;
3. enabled constructing and executing of heterogeneous pipelines involving a large number of datasets and multiple types of data processing tools;
4. the overall usability of grid resources is improved by the intuitive graphical interface offered by the LONI Pipeline environment, and
5. interim results from user-specified modules can be interactively displayed (interactive outcome checking).

### 2.5 Virtualization

We virtualized the LONI Pipeline cluster for outside distribution using a suite of open-source neuroimaging tools. That Pipeline Virtual Machine (VM) infrastructure provides end-users with the latest stable pre-compiled and pre-installed open source neuroimaging applications. The resulting VM, referred to as the *Pipeline Neuroimaging Virtual Environment* (PNVE), is a completely self-contained execution environment that can be run locally or on another grid computing environment, such as Amazon's Elastic Compute Cloud (EC2, http://aws.amazon.com/ec2/). Because the PNVE application environment tightly mirrors that of the LONI grid, users with access to LONI's resources gain the flexibility to seamlessly switch between local or remote execution. PNVE version 1.0 (http://pipeline.loni.ucla.edu/downloads/pnve) is based on Ubuntu (www.ubuntu.com) and VMware (www.vmware.com/) software technologies and includes many major open-source neuroimaging tools. We include instructions for local execution, converting to other VM formats (e.g. VirtualBox), and converting to an EC2-friendly format, as well as the steps required to run the PNVE within the EC2 environment. PNVE provides automated installation of neuroimaging applications as well as updates of Pipeline modules and workflows for automatically building the virtual images.

### 2.6 LONI Pipeline Data Provenance

In neuroimaging studies, data provenance, or the history of how the data were acquired and subsequently processed, is often discussed but seldom implemented [24]. Recently, several groups have proposed provenance challenges in order to evaluate the status of various provenance models [25]. An example of such provenance challenge is collecting provenance information from a simple neuroimaging workflow [12], [26] and documenting the system's response over repeated executions with varying data. It is difficult to provide systematic, accurate and comprehensive capture of provenance information with minimal user intervention. The processes of data provenance and curation are significantly automated via the LONI Pipeline. Each dataset has a provenance file (*.prov) that is automatically updated by the LONI Pipeline, based on the protocols used in the data analysis. This data processing history reflects the steps that a dataset goes through and provides a detailed record of the types of tools, versions, platforms, parameters, control and compilation flags. The data provenance can be imported and exported by the LONI Pipeline, which enables utilization internally by other Pipeline workflows or by external resources.

Provenance can be used for determining data quality, for result interpretation, and for protocol interoperability [26], [27]. It is imperative that the provenance of neuroimaging data be easily captured and readily accessible [12]. For instance, increasingly complex analysis workflows are being developed to extract information from large cross-sectional or longitudinal studies in multiple sclerosis [28], Alzheimer's disease [29], autism [30], depression [31], schizophrenia [32], and studies of normal populations [33]. The implementation of the complex workflows associated with these studies requires provenance-based quality control to ensure the accuracy, reproducibility, and reusability of the data and analysis protocols.

We designed the provenance framework to take advantage of context information that can be retrieved and stored while data is being processed within the LONI Pipeline environment [24]. Additionally, the LONI Provenance Editor is a self-contained, platform-independent application that automatically extracts provenance information from image headers (such as a DICOM images) and generates an XML data provenance file with that information. The Provenance Editor (http://www.loni.ucla.edu/Software/ProvenanceEditor) allows the user to edit the metadata prior to saving the provenance file, correcting inaccuracies or adding additional information. This provenance information is stored in .*prov* files, XML formatted files that contain the metadata and processing provenance and follows the XSD definition. Then the data provenance is expanded by the LONI Pipeline to include the analysis protocol, the specific binaries used for analysis, and the environment that they were run in. The LONI Pipeline dramatically improves compliance by minimizing the burden on the provenance curator. This frees the user to focus on performing neuroimaging research rather than on managing provenance information.

## Results

In this section, we describe three specific applications that illustrate the utilization of the LONI Pipeline for construction and validation of neuroimaging analysis protocols, for assembling of computational meta-algorithms, and for integration of tools and data resources available through different workflow environments.

### 3.1 Neuroimaging Applications

#### Tensor-Based Morphometry (TBM)

**Figure 4:**
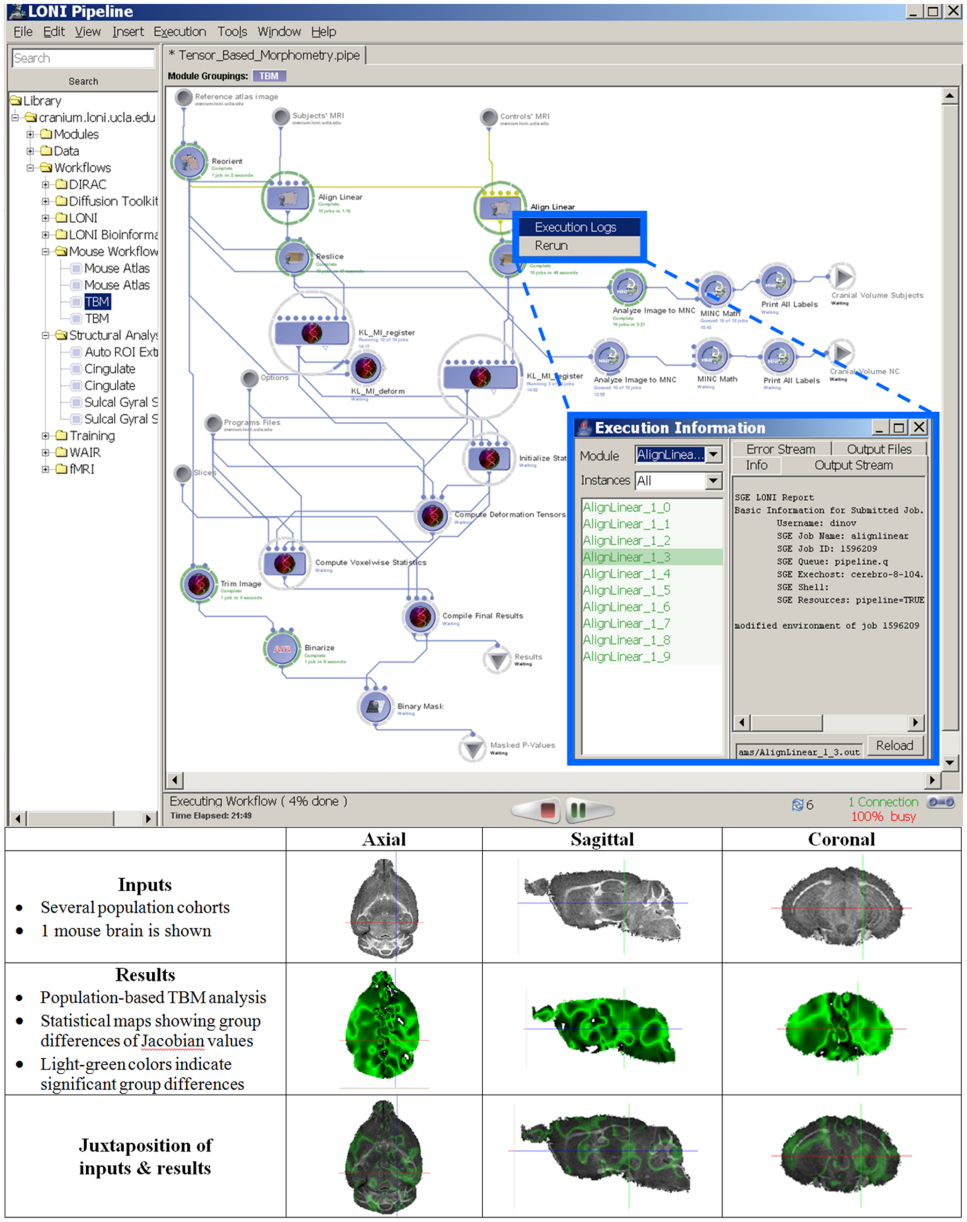
The LONI tensor-based morphometry (TBM) pipeline workflow. This pipeline demonstrates the interoperability of several independently-developed computational neuroscience tools, management of grid-distributed jobs (different subjects and independent operations are executed in parallel), and the interactive process-monitoring framework for exploring the state of the entire execution workflow, as well as each individual module and input case.

Contemporary neuroimaging studies often require multiple heterogeneous processing steps on large datasets. In this context, *heterogeneous* refers the diversity of the tools which are interoperating within a common pipeline workflow – this includes types of resources (data, tools, services), types of programming languages, compilers, and configurations, as well as differences in tool design and implementation. The Pipeline allows the integration of such disparate tools developed by different groups and for different purposes. There are anatomical [34], [35], functional [36], [37] and mixed [38], [39] forms of imaging and physiological measurements [40], as well as cyto- and chemo-architecture [41], gene localization [42], and phenotype-genotype interaction studies [43]. Virtually all neuroimaging investigations are computationally intensive and incorporate a mixture of manual and automated processing. For instance, a tensor-based morphometry [44] pipeline involves several distinct and independent software resources, as shown in **Figure 4**. Data [45] and tools from several software packages [46], [47], [48] are employed in this heterogeneous pipeline workflow. The LONI Pipeline already includes dozens of heterogeneous workflows in its core resource library. Complete analysis workflows are available via the LONI Pipeline client/server application as well as via a web-service from the LONI Pipeline web-page (http://pipeline.loni.ucla.edu/). The LONI Pipeline Graphical User Interface (GUI) allows users to dynamically describe and save new data, resources and services modules, and construct new heterogeneous pipelines as workflows integrating these modules.

#### Global Shape Analysis (GSA)

**Figure 5:**
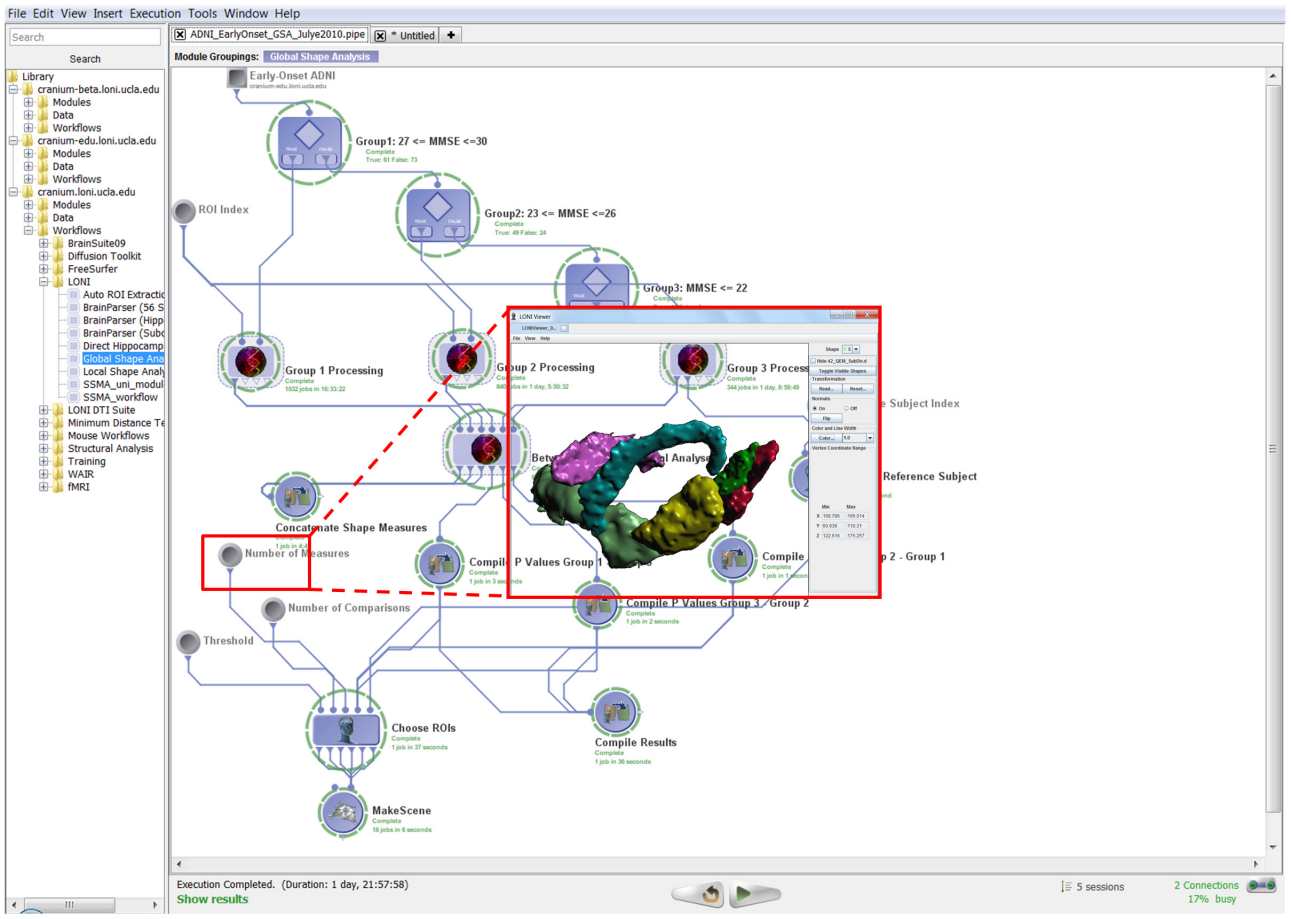
The global shape analysis (GSA) pipeline workflow. This workflow illustrates the protocol for construction of study-designs, automated ROI parsing, volumetric and shape measure calculations and between cohorts statistical analysis. 134 ADNI [1] subjects are used in this study representing three independent cohorts classified by their Mini Mental State Exam (MMSE) scores - 18 Alzheimer's disease (AD) patients, 49 Mild Cognitive Impairment (MCI) subjects and 61 asymptomatic normals (NC). The workflow computes 6 global shape measures for each of 56 automatically extracted regions of interest (ROIs) for all subjects [2]. Then, between-cohort statistics of these shape measures are calculated for each region of interest. The output of this workflow includes 18 3D scenes (3 possible group comparisons and 6 different shape measures). Each 3D scene contains only the ROI models of the regions where of the pair of cohorts showed statistically significant global shape differences. In this study-design, there are a total of 18 3D scene outputs reflecting the 3 possible pairs of group analyses (contrasts) comparing two cohorts (AD-NC, NC-MCI, AD-MCI) and the 6 different shape measures (mean-curvature, surface area, volume, shape-index, curvedness and fractal dimension). This workflow completed in about 46 hours on a small 56-node cluster and included a total of 3,209 jobs. The insert image only shows the 3D scene result for the ROIs which had statistically significant difference in mean-curvature between AD and NC subjects. For this comparison and shape measure, the resulting ROIs and their (labels) included right insular cortex (102), right middle orbitofrontal gyrus (30), right postcentral gyrus (42), left cingulated gyrus (121), left gyrus rectus (33) and left postcentral gyrus (41).

Robust automated detection, modeling and analysis of regional brain anatomy are significant challenges in large-scale neuroimaging studies. We designed a heterogeneous Pipeline workflow which employs the new study-design mechanism and a diverse set of volume and shape processing tools to address these challenges. This automated protocol takes the imaging data (Magnetic Resonance Imaging, MRI, T1-weighted volumes) and non-imaging (subject demographics) metadata for a collection of subjects, obtains a signature vector of global volumetric and shape-based measures for each region of interest (ROIs) and each subject, and conducts statistical analyses to identify regional differences between three subject cohorts (Alzheimer's disease (AD), asymptomatic normal controls (NC) and mild cognitive impairment (MCI) subjects), **Figure 5**. This analysis was based on 134 ADNI [64] subjects representing three independent cohorts classified by their Mini Mental State Exam (MMSE) scores [49] —18 Alzheimer's disease (AD) patients, 49 Mild Cognitive Impairment (MCI) subjects and 61 asymptomatic normals (NC). The workflow computes 6 global shape measures (mean-curvature, surface area, volume, shape-index, curvedness and fractal dimension) for each of 56 automatically extracted regions of interest (ROIs) for all subjects [65]. Then, between-cohort statistics of these shape measures are calculated for each region of interest. The workflow output consists of 18 3D scenes (3 possible group comparisons and 6 different shape measures). Each 3D scene contains only the ROI models of the regions where of the pair of cohorts showed statistically significant global shape differences. The insert in **Figure 5** only shows one 3D scene result for the ROIs which had statistically significant difference in mean-curvature between AD and NC subjects. For this specific comparison, the resulting ROIs included right (insular cortex, middle orbitofrontal gyrus and postcentral gyrus) and left (cingulated gyrus, gyrus rectus and postcentral gyrus) hemispheric regions.

### 3.2 Development of Meta-Algorithms

**Figure 6:**
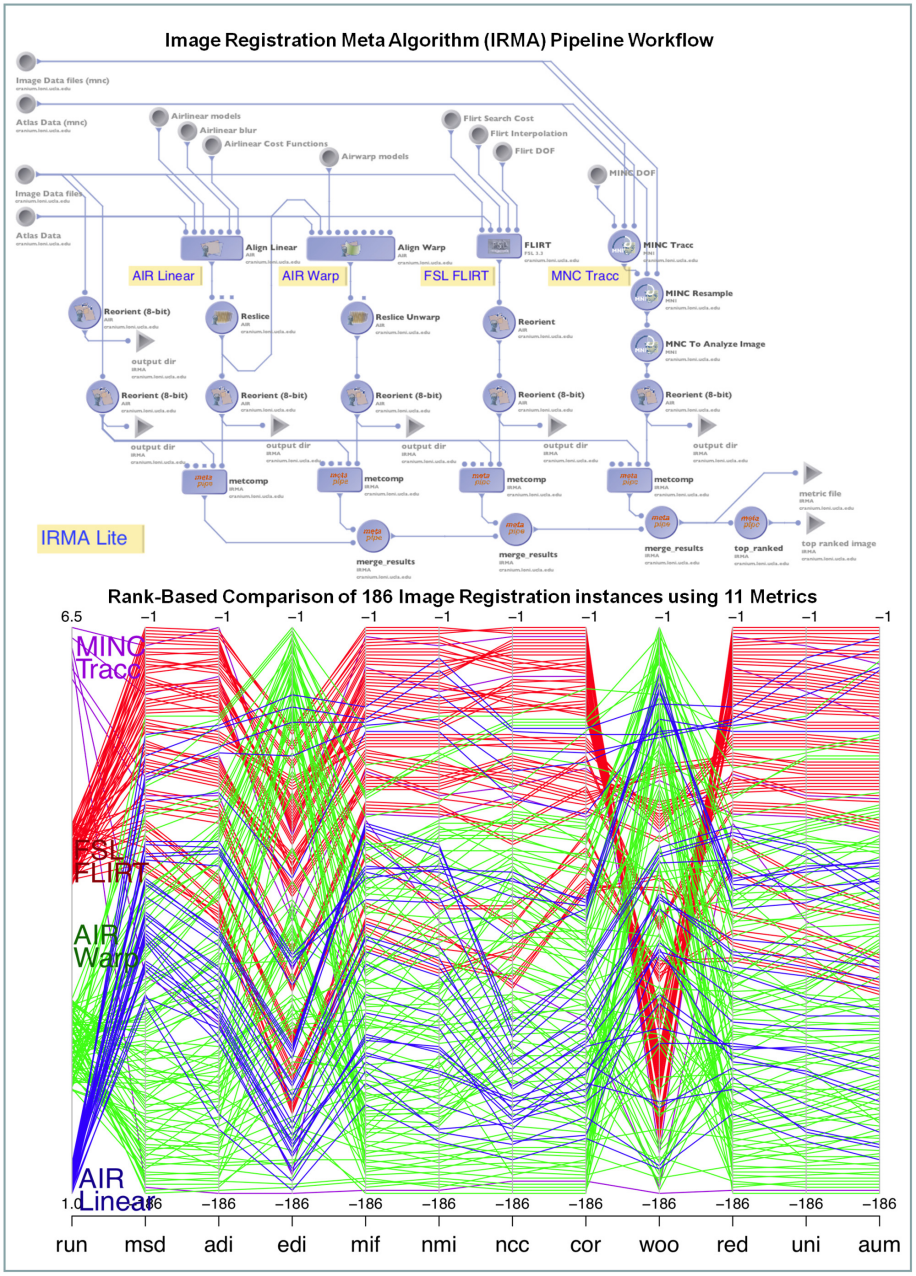
Pipeline meta-algorithm developments. (*Top Panel*) This figure shows the LONI Pipeline implementation of the image registration meta-algorithm (IRMA). The four different registration algorithms AIR Linear, AIR Warp, FLIRT and MINC Tracc are present as individual nodes in this pipeline. Parameter sets specifying altogether 186 different runs for these algorithms are stored as editable lists within the LONI Pipeline environment. (*Bottom Panel*) Parallel-coordinates plot showing the rank-transformed metrics for one input image. Eleven distance metrics were employed to evaluate all 186 alignment instances. The results of the IRMA analysis illustrate that the FLIRT family performed consistently better than the other alignment methods for this input volume. The EDI (Entropy of Difference of Intensities) and the Woo (Woods' coefficient) metrics disagree somewhat with the other 9 metrics.

Meta-algorithms pool the results of many other algorithms implementing for the same task in several ways [13]. The basic principle underlying all meta-algorithms is to achieve higher robustness, improve reliability and accuracy than any of the individual algorithms they engage. In essence, meta-algorithms facilitate an ensemble framework that allows different (or similar) algorithms to perform the same task and can be considered mini-max optimal [50]. Meta-algorithms focus on “consistency” in the decision-making, avoiding intrinsic assumptions made by the individual algorithms using a battery of performance measurements applied on the outputs of the algorithms in a consensual and yet independent fashion. One example of a meta-algorithm that we recently implemented within the LONI Pipeline environment is the Image Registration Meta-Algorithm (IRMA) [51]. IRMA is a method for combining results of several different image registration algorithms. The current IRMA implementation employs 4 registration algorithms and assesses the warped images with a battery of 11 distance metric measurements using sophisticated sampling and robust statistical estimates. IRMA uses the data mining technique of dimensionality reduction method to choose a ‘best’ registration. Brain image registration is the process of aligning brain images to obtain a correspondence between a series of subjects that allows finding homologous anatomical landmarks in all subject. Similarity of brain images is measured by how close, in some metric, the registration method aligns the images to optimize the distance between them [47]. In our IRMA validation protocol employs 186 registration instances derived from a set of 4 warping families – Linear and Nonlinear AIR registration tools [47], FLIRT [18], and MINC Tracc [52]. In **Figure 6**, the top panel demonstrates the integrated LONI Pipeline representing the IRMA meta-algorithm. The ranks of all 186 registration instances (from 4 different alignment families) based on volumetric MRI data are shown in the ranked parallel coordinates plot in the bottom panel. For this particular input image, the result of IRMA suggests that the FLIRT registration family is better than the other registration algorithms, despite some disagreements occurring in the EDI (Entropy of difference in Intensity, [53]) and the Woo (Woods' coefficient, [47]) metrics.

## Discussion

Interactive workflow environments for automated data analysis are critical in many research studies involving complex computations and large datasets [54], [55], [56], [57]. There are three distinct necessities that underlie the importance of such graphical frameworks for management of novel analysis strategies – high data volume and complexity, sophisticated study protocols and demands for distributed computational resources. These three fundamental needs are evident in most modern neuroimaging, bioinformatics and multidisciplinary studies.

The LONI Pipeline environment provides distributed access to various computational resources via its graphical interface. The ability of investigators to share, integrate, collaborate and expand resources increases the statistical power in studies involving heterogeneous datasets and complex analysis protocols. New challenges that emerge from our increased ability to utilize computational resources and hardware infrastructure include the need to assure reliability and reproducibility of identically analyzed data, and the desire to continually lower the costs of employing and sharing data, tools and services. The LONI Pipeline environment attempts to address these difficulties by providing secure integrated access to resource visualization, databases and intelligent agents (e.g., keyword or phrase-based automated workflow generators).

The LONI Pipeline already has been used in a number of neuroimaging applications including health [35], disease [58], [59], [60], [61], animal models [42], [62], volumetric [63], [64], functional [65], [66], [67], shape [32], [66] and tensor-based [68], [69] studies, and meta-algorithm developments [51]. The LONI Pipeline infrastructure improved consistency, reduced development and execution times, and enabled new functionality and usability of the analysis protocols designed by expert investigators in all of these studies. Perhaps the most powerful feature provided by the LONI Pipeline environment is the ability to quickly communicate new protocols, data, tools and service resources, findings and challenges to the wider community.

The main LONI Pipeline page (http://pipeline.loni.ucla.edu) provides links to the forum, support, video tutorials and usage. There are examples demonstrating how to describe individual modules and construct integrated workflows. Version information, download instructions and server/forum account information is also available on this page. There are example pipeline workflows and the XSD schema definition for the .*pipe* format used for module and workflow XML description. Users may either install Pipeline servers on their own hardware systems, or, they may use some of the available Pipeline service resources. Users may obtain accounts on the LONI grid by going to http://www.loni.ucla.edu/Collaboration/Pipeline/Pipeline_Download.jsp. There are on average 140–200 monthly downloads of the LONI Pipeline environment.

In general, some practical difficulties in validating new LONI Pipeline workflows may be caused by unavailability of the initial raw data, differences of hardware infrastructures or variations in compiler settings and platform configurations. Such situations require analysis workflow validation of the input, output and state of each module within the pipeline workflow. Further LONI Pipeline validation would require comparison between synergistic workflows that are implemented using different executable modules or module specifications. For example, one may be interested in comparing similar analysis workflows by choosing different sets of imaging filters, reconfiguring computation parameters or manipulating the resulting outcomes, e.g., file format, [15]. Such studies contrasting the benefits and limitations of each resource or processing workflow aid both application developers and general users in the decision of how to design and utilize module and pipeline definitions to improve resource usability.

A significant challenge in computational neuroimaging studies is the problem of reproducing findings and validating analyses described by different investigators. Frequently, methodological details described in research publications may be insufficient to accurately reconstruct the analysis protocol used to study the data. Such methodological ambiguity or incompleteness may lead to misunderstanding, misinterpretation or reduction of usability of newly proposed techniques. The LONI Pipeline mediates these difficulties by providing clear, functional and complete record of the methodological and technological protocols for the analysis.

The new neuroimaging study-design, data-management, virtualization environment is available for download, testing and validation via the LONI Pipeline web-page http://pipeline.loni.ucla.edu. This URL also contains links to forum, Q/A, user guide, screencasts and video tutorials. Users can download and install the Pipeline as a client or a server on Windows, Linux or Mac platforms. Future improvements will include extension of the available image processing filters, addition of new complete pipeline graphical workflows, extending the available backend grid plugin interfaces, and providing metadata augmentation functionality. We are also working on several new features of the LONI Pipeline including a webservice-based client interface, direct integration with external resource archives (e.g., http://neuinfo.org) and interface enhancements using intelligent plugin components.

The Pipeline support pages include user-guides, screencasts, training handbook and example workflows that are useful to novice and expert users alike (http://pipeline.loni.ucla.edu/support/).
